# Multimodal noncontact atomic force microscopy and Kelvin probe force microscopy investigations of organolead tribromide perovskite single crystals

**DOI:** 10.3762/bjnano.9.161

**Published:** 2018-06-07

**Authors:** Yann Almadori, David Moerman, Jaume Llacer Martinez, Philippe Leclère, Benjamin Grévin

**Affiliations:** 1 Université Grenoble Alpes, CNRS, CEA, INAC-SyMMES, 38000 Grenoble, France,; 2 Laboratory for Chemistry of Novel Materials, Center of Innovation and Research in Materials & Polymers (CIRMAP), University of Mons, Place du Parc 20, B7000 Mons, Belgium

**Keywords:** carrier lifetime, ion migration, Kelvin probe force microscopy (KPFM), noncontact atomic force microscopy (nc-AFM), organic–inorganic hybrid perovskites, photostriction, single crystals, surface photovoltage (SPV), time-resolved surface photovoltage

## Abstract

In this work, methylammonium lead tribromide (MAPbBr_3_) single crystals are studied by noncontact atomic force microscopy (nc-AFM) and Kelvin probe force microscopy (KPFM). We demonstrate that the surface photovoltage and crystal photostriction can be simultaneously investigated by implementing a specific protocol based on the acquisition of the tip height and surface potential during illumination sequences. The obtained data confirm the existence of lattice expansion under illumination in MAPbBr_3_ and that negative photocarriers accumulate near the crystal surface due to band bending effects. Time-dependent changes of the surface potential occurring under illumination on the scale of a few seconds reveal the existence of slow ion-migration mechanisms. Lastly, photopotential decay at the sub-millisecond time scale related to the photocarrier lifetime is quantified by performing KPFM measurements under frequency-modulated illumination. Our multimodal approach provides a unique way to investigate the interplay between the charges and ionic species, the photocarrier-lattice coupling and the photocarrier dynamics in hybrid perovskites.

## Introduction

Organic–inorganic hybrid perovskites (RMX_3_, where R = methylammonium or formamidinium, M = Pb or Sn, and X = halogen) have become a new platform for the development of next-generation light harvesting and optoelectronic devices in the past years [1]. Indeed, they exhibit an exceptional combination of optoelectronic properties, including a direct band gap, high absorption coefficient, large and balanced carrier mobility, high diffusion length, long carrier lifetime and high photoluminescence quantum yield. Within a few years of their discovery, these materials were successfully used to develop photovoltaic cells [2] with power conversion efficiencies exceeding 20% and several kinds of optoelectronic devices, including efficient light-emitting diodes [3], laser devices [4] and high-gain photodetectors [5].

Recently, Kelvin probe force microscopy (KPFM) has been used to investigate the impact of grain boundaries (GBs) on the internal electric field distribution and photocarrier recombination mechanisms in polycrystalline perovskite thin films [6–7]. However, considering the results of earlier works shows that it is sometimes difficult to draw definitive conclusions about the detrimental (or beneficial) impact of the GBs on the photocarrier transport on the sole basis of KPFM data. This uncertainty is largely due to the contributions of the ionic species to the surface potential contrasts [6,8–10]. Time-resolved measurements have especially shown that intra-grain ion-migration mechanisms [9] can significantly impact the surface potential probed by KPFM. It is now clear that a complex interplay exists between the charge carrier populations, traps, and mobile ions. Despite all the progress made, interpreting the surface potential (SP) and surface photovoltage (SPV) contrasts recorded by KPFM on polycrystalline lead halide perovskite thin films remains a difficult task.

Over the last few years single crystals [11–12] have constituted an interesting alternative for basic research on hybrid perovskites. Thanks to the absence of grain boundaries (and noncrystalline domains) they can be advantageously used to probe the intrinsic material optoelectronic properties. Performing KPFM measurements on single crystals may therefore facilitate the interpretation of the SP and SPV data. Moreover, scanning probe microscopy measurements may help in distinguishing the properties of the bulk from the surface [13]. However, so far, KPFM investigations of hybrid perovskite single crystals remain rather limited [14–15].

Another important point to consider is the existence of photostriction effects, which have actually been observed in MAPbI_3_ and MAPbBr_3_ single crystals [16–17]. In the most general terms, photostriction can be defined as a nonthermal sample deformation under illumination. This effect is widely documented for ferroelectrics, polar and nonpolar semiconductors, and organic polymers, and it differs in origin depending on the class of material under consideration [18]. For instance, in the case of ferroelectric oxides, it originates indirectly from the superposition of photovoltaic and converse piezoelectricity effects (we refer the reader to review articles [18] for a more comprehensive introduction to the field of photostrictive materials). The photostriction observed by a few teams in organolead trialides is most probably related to the photovoltaic effect [16–17] and strong photon–lattice coupling [16], but its exact mechanism remains to be clarified.

In principle, the photostrictive response of any material can be simply probed by recording the height variation of an AFM tip as a function of the illumination state [16]. However, one can arguably invoke the existence of artefacts prone to affect this kind of measurement [17]. In recent work, Zhou et al. carried out a comprehensive series of experiments on MAPbI_3_ single crystals (and thin films), providing strong evidence that the height changes probed by AFM under illumination originate mainly from the intrinsic material deformation [16]. More precisely, thanks to a rigorous experimental protocol, they demonstrated that it is possible to discriminate between the intrinsic material deformation and the extrinsic effects related to the AFM cantilever light-induced perturbation and thermal relaxation. In addition, by monitoring the sample temperature and analyzing the temporal evolution of the height change probed by AFM they were able to rule out possible contributions from the thermal expansion of the sample (we refer the reader to [16] and the related supporting information for more details).

Now, the question that presents itself is whether the photostriction can influence the results of SPV measurements by KPFM. In addition, valuable information about the light–matter interaction process may be gained by simultaneously measuring the light-induced SP and lattice changes. Lastly, the tip–sample height measured in dynamic AFM is prone to be affected by variations of the electrostatic forces, which in turn, vary as a function of the illumination state of the photovoltaic sample. Thus, for accurate photostrictive measurements, it is highly desirable to nullify (or a least minimize) the electrostatic forces by using an active KPFM compensation potential loop.

In this work, the photovoltaic and optomechanical properties of a methylammonium lead tribromide (CH_3_NH_3_PbBr_3_, also referred to as MAPbBr_3_) single crystal are investigated by noncontact AFM (nc-AFM) combined with KPFM. MAPbBr_3_ has been selected for these experiments since its absorption band edge [12] falls well below the wavelength of the AFM light source (840 nm for the Omicron VT-AFM setup used here). A specific protocol allowing simultaneous recording of the spectroscopic curves as a function of time for the AFM tip height relative to the surface (*z*(*t*)) and of the surface potential (SP(*t*)) during pulsed illumination sequences is implemented. The AFM/KPFM signals are moreover investigated as a function of the optical excitation wavelength and fluence (with an optical power variation covering several decades). The analysis of the full data set allows the confirmation that the height and SP variations under illumination originate from intrinsic photostriction and photovoltaïc effects, respectively. Furthermore, we show that the surface photovoltage decay can be probed by performing KPFM measurements under frequency-modulated illumination. These results establish that nc-AFM/KPFM can be effectively used to investigate both the photocarrier dynamics and the photon–lattice coupling in organic–inorganic hybrid perovskites.

## Methods

### Sample preparation

Methylammonium lead tribromide single crystals (millimeter- to centimeter-sized) were grown from *N*-dimethylformamide (DMF) solution at constant temperature. In this process [12] CH_3_NH_3_Br and PbBr_2_ precursors are used that are soluble in DMF at room temperature, and the crystallization occurs between 90 °C and 100°C due to inverse temperature solubility. The single crystal investigated under ultrahigh vacuum (UHV) was fixed on a stainless steel sample UVH holder with a compatible electrically conductive silver epoxy paste (EPO-TEK E4110), which was cured at room temperature (RT) over 24 hours. The sample was subsequently cleaved with a scalpel just before being introduced in the load-lock of the VT-AFM (after cleavage, the sample thickness was estimated to be on the order of 1 mm).

### Noncontact AFM and Kelvin probe force microscopy

The nc-AFM experiments were carried out with an Omicron VT-AFM setup in ultrahigh vacuum (UHV) at room temperature (RT) with in situ annealed Pt/Ir-coated silicon cantilevers (EFM, Nanosensors, resonance frequency in the 45–115 kHz range). Topographical imaging was performed in frequency modulation mode (FM-AFM) with negative frequency shifts of a few Hz and vibration amplitudes of a few tens of nanometers. KPFM measurements were carried out in single-pass mode under frequency modulation (FM-KPFM) with the modulation bias, *V*_AC_ (typically 0.5 V peak-to-peak at 1200 Hz), and the compensation voltage, *V*_DC_, applied to the cantilever (tip bias *V*_tip_ = *V*_DC_). The contact potential difference (CPD = *W*_tip_ − *W*_sample_, where *W* is the work function divided by the elementary charge in absolute value) is thus the opposite of *V*_DC_ (more details about these polarity conventions can be found in [19]). In the following, the KPFM data are presented as compensation bias (*V*_tip_ = −CPD) images (also called the KPFM potential or surface potential images for simplicity).

### Sample illumination

External fiber-coupled laser sources (Omicron Laserage GmBH, LuxX modules operated at 405, 515 and 685 nm or a PhoxXplus unit operated at 515 nm) were used for sample illumination (front side geometry, i.e., from the top) through an optical viewport of the UHV AFM chamber. For each measurement, the optical power *P*_opt_ (defined per unit of surface by taking into account the laser beam diameter) and wavelength λ are indicated in the corresponding figure caption. Note that the optical beam makes a 30° angle with respect to the sample plane (for simplicity *P*_opt_ was given at the output of the laser module fiber, without correction for the incidence angle).

The modules were calibrated prior to the KPFM measurements by measuring the power at the fiber output with a power meter. With these laser systems, the power regulation is inaccurate within a few percent of the maximum operating power (typically a few tens of milliwatts for our modules). A series of optical density filters (OD, with 1, 2 and 4 attenuation factors in log scale) were used to attenuate the illumination power, which allowed variation of the illumination power over several decades. For each optical density filter, the exact attenuation factor was calibrated at 405, 515 and 685 nm prior to the experiments. The curves of the photo-physical parameters (SPV and photostriction) as a function of the illumination power were reconstructed by merging the data acquired with different densities. Different symbols (indicated in the figure captions) corresponding to each density are used hereafter to plot the curves.

### Spectroscopic and time-resolved measurements

An arbitrary waveform generator (AWG, Keysight 33622A) was used to operate the laser sources in digital modulation mode. Logic signals generated by the scanning probe microscope controller were used to trigger the generation of illumination pulse sequences by the AWG operated in “burst” mode. Spectroscopic data were acquired by simultaneously recording the temporal evolution of the surface potential (SP(*t*)) and the AFM tip height (*z*(*t*)) as the sample is subjected to an illumination sequence. Time-resolved measurements of the sub-millisecond SPV decays were performed by recording spectroscopic curves of the average surface potential as a function of the modulation frequency of the illumination source. By analyzing the dependency of the average potential with respect to the modulation frequency, it is possible to extract time constants characterizing the photopotential decay dynamics between the light pulses. More information about KPFM operations under frequency-modulated illumination (FMI-KPFM) can be found in our previous report [20].

## Results and Discussion

**Figure 1:**
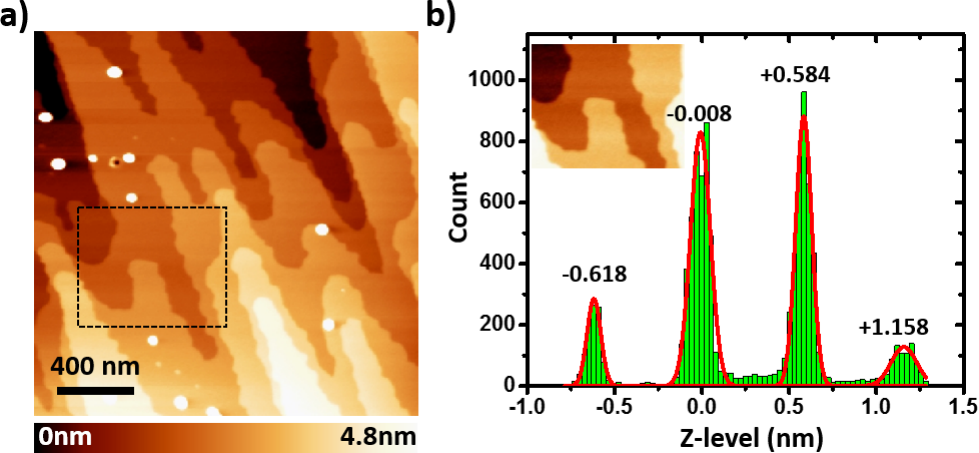
(a) nc-AFM topographic image (2 × 2 μm) of the MAPbBr_3_ single crystal surface. The dotted rectangle highlights the area used to calculate the *z*-level histogram. (b) Height histogram of the image in the inset (numeric zoom from the image in (a)). The red line shows the result of a multiple Gaussian peak fit. The average step height deduced from the peak positions is equal to 0.59 ± 0.01 nm.

The topographic nc-AFM images of the single crystal surface (Figure 1a) feature smooth terraces a few hundreds of nanometers wide. The step height deduced from *z*-level histograms (Figure 1b) is exactly equal to the cubic perovskite unit cell (0.59 nm for MAPbBr_3_ [1,21]). This confirms that the surface investigated corresponds to the (100) plane of the MAPbBr_3_ crystal. Several features appearing as white spots in the topographic images indicate moreover the likelihood of defects, which could be attributed to surface contamination upon exposure to ambient atmosphere during the cleaving process. Alternatively, one may also hypothesize that intrinsic defects are formed during the solution process crystal growth. Addressing the origin of these defects is beyond the scope of the current work, and will require development of in situ cleaving facilities (enabling discrimination between extrinsic surface contamination and intrinsic defect formation). Here, our primary goal is to check if the surface photovoltage and crystal photostriction can be simultaneously and reliably probed by nc-AFM/KPFM. The discussion will be thus focused on the analysis of the single crystal response on the basis of spectroscopic curves acquired in point mode (i.e., at selected locations on the surface).

**Figure 2:**
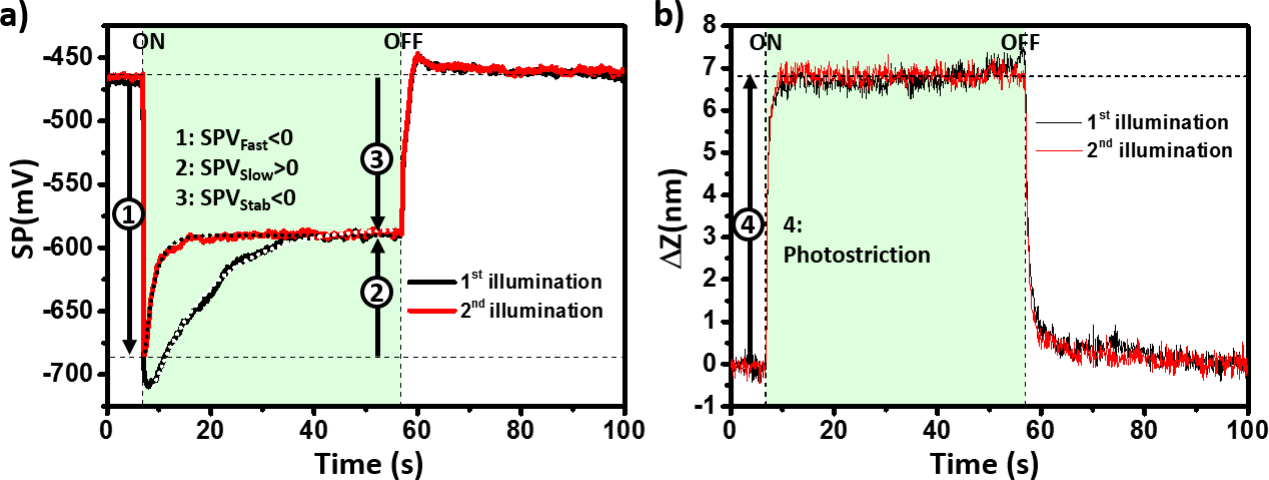
Plots of the (a) KPFM surface potential (b) and the tip height change relative to its initial position as a function of time during two successive illumination sequences (λ = 515 nm, *P*_opt_ = 2.95 mW/cm^2^). The arrows labelled 1, 2, 3 in (a) and 4 in (b) highlight the fast (1), slow (2) and stabilized (3) components of the surface photovoltage and the photostriction signal (4), respectively. The dotted curves in (a) show the results of curve adjustments with functions based on a single exponential, yielding time constants for the SPV dynamics of 11.4 s and 2.1 s during the 1st and 2nd illumination sequences, respectively.

Figure 2a,b shows the KPFM surface potential (SP) and the tip height curves recorded during two successive single-pulse illumination sequences separated by a time interval of a few tens of seconds (at an excitation wavelength of 515 nm and with an optical power of 2.95 mW/cm^2^). First, we note that the SP exhibits a quasi-instantaneous response (at the timescale of the KPFM regulation loop integration time, which was set to a few tens of ms for these experiments) in the form of a negative shift of ≈240 mV after switching the light pulse on. This fast change is followed by a slower evolution and a subsequent stabilization of the SP under illumination at a timescale of a few tens of seconds. The surface photovoltage (SPV) at equilibrium (or “stabilized SPV”, SPV_Stab_) is therefore equal to the sum of a negative and a positive term, which will be referred to hereafter as “fast” and “slow” SPV components (SPV_Fast_ and SPV_Slow_, see Figure 2a) with regards to their different photoresponse dynamics. After switching the light off, the SP displays a fast positive shift followed by a slow stabilization towards its initial level. Remarkably, the tip height also displays a fast photoresponse, but shows almost no noticeable evolution under continuous illumination at this optical power. In other words, the maximum height photoresponse is quasi-instantaneous (at the time scale of our measurement) and does not scale with the illumination time. Consistent with the conclusions of the former work by Zhou et al. [16], this strongly supports the idea that the “fast” cantilever height photoresponse originates from an intrinsic photostriction effect (and not from a thermally induced sample dilatation).

Another significant difference is that the *z*-curves recorded during the first and the second illumination sequences are perfectly identical; in turn, the SP stabilizes more quickly under illumination during the second sequence. Time constants characterizing the SPV evolution under illumination can be deduced from the curve adjustments (shown as the dotted curves in Figure 2a) with single exponential based functions. They are equal to 11.4 s and 2.1 s for the first and second illumination sequence, respectively. Actually, complementary measurements performed by applying a series of successive pulses (see Figure S1 in Supporting Information File 1) demonstrate that the SP stabilization time constant under illumination does not evolve further after the second illumination pulse. In the following, the methodology used to calculate the SPV values consists of applying a first “initialization” light pulse. The data are then calculated from curves that are recorded during subsequent illumination sequences.

The different SP and height photoresponses already indicate that the surface photovoltage and photostriction effect probed in our experiment do not result from crosstalk between the *z* and KPFM regulation loops. In addition, two simple tests have been carried out to definitely exclude the existence of artefacts (see Figures S2 and S3 in Supporting Information File 1). First, the influence of the light pulse on the *z* regulation has been checked by recording spectroscopic curves of the frequency shift with the AFM tip in full backward position (i.e., retracted 1 μm away from the sample surface and kept at a fixed position with the topographic regulation disengaged). By comparing the frequency detuning induced by the light pulse with curves of the tip height (recorded in the interaction with an active regulation) as a function of the frequency set point (Figure S2, Supporting Information File 1), it can be simply shown that the cantilever detuning can at maximum (i.e., for the largest optical power applied in this study) induce a *z*-shift of 0.15 nm. Secondly, spectroscopic curves were acquired under the same illumination conditions and with the same cantilever on a highly oriented pyrolytic graphite (HOPG) substrate (Figure S3, Supporting Information File 1). The surface potential displays no shift under illumination (which also confirms the absence of any carrier photogeneration due to the cantilever tip itself), and the fast component of the *z* photoresponse is negligible compared to the one measured on the MAPbBr_3_ single crystal with the same fluence. This reinforces the conclusion that the fast component of the *z* photoresponse detected on the MAPbBr_3_ crystal does not originate from a thermal expansion effect. Note here that the HOPG substrate displays a thermal expansion coefficient [22] in the out-of-plane direction close to that of the MAPbBr_3_ crystal [23] and that both samples are relatively similar in terms of size (0.5 mm thick for the HOPG vs ≈1 mm for the MAPbBr_3_ crystal).

These comparative measurements on HOPG show that at high fluence, the thermal detuning of the cantilever can induce a slow evolution of the *z* level under illumination and a subsequent slow return to equilibrium in dark conditions. Nevertheless, this extrinsic *z*-change has no impact on the SP measurement, as demonstrated by the data acquired on the HOPG substrate. Finally, both the SPV and the photostrictive response show a clear dependence as a function of the photon energy (as shown in Figure S4, Supporting Information File 1). For equivalent optical powers, much smaller height variations and SP shifts are observed when the wavelength falls below the MAPbBr_3_ bandgap (*E*_G_ ≈ 2.2eV [12]). This confirms that the measured height changes originate from the intrinsic photostriction of the MAPbBr_3_ crystal. However, an almost identical photoresponse is observed under 405 nm and 515 nm illumination, which seems different from the case of MAPbI_3_ (for which a wavelength-dependent photostriction was observed [16] above the bandgap). Here, it is noteworthy that the wavelength of our green laser falls within an absorption peak due to a strong excitonic transition [24–25]. Further measurements at intermediate wavelengths (currently unavailable in our setup) would be necessary to draw a definitive conclusion about the wavelength dependency of the photoresponse above the bandgap.

**Figure 3:**
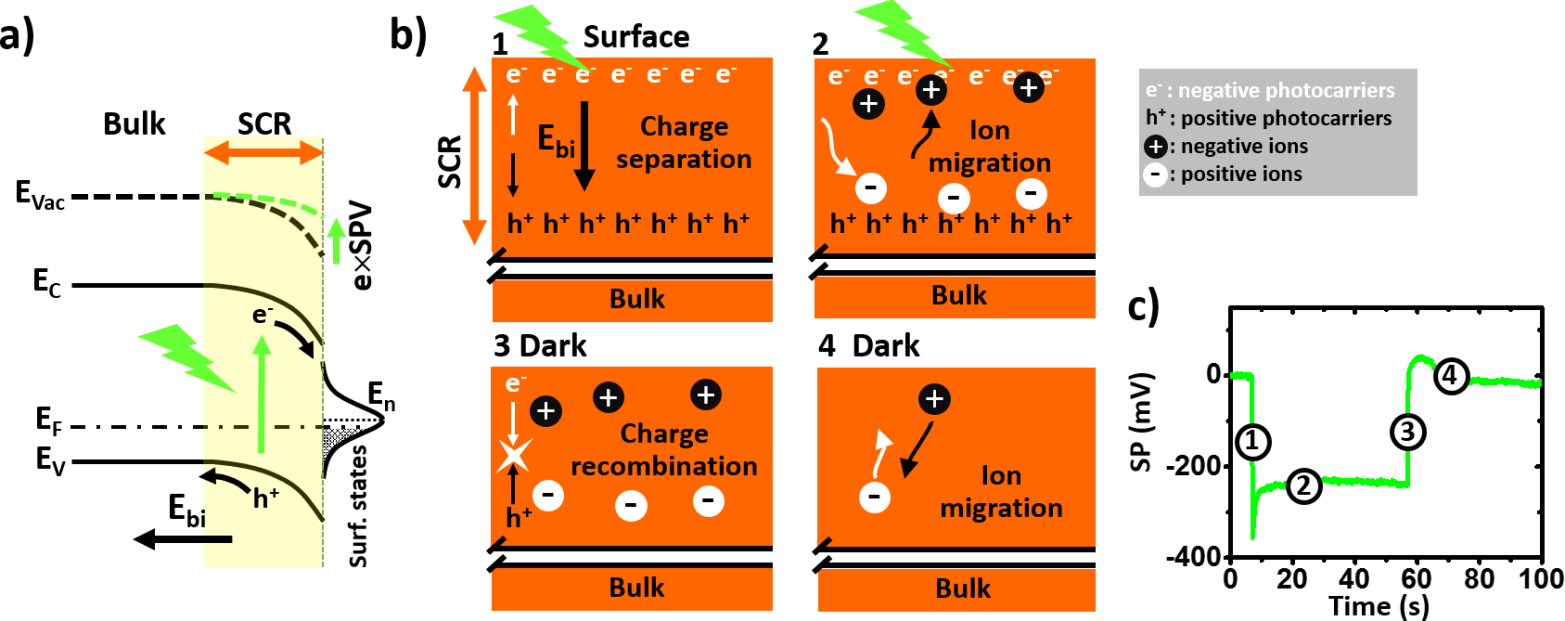
(a) Scheme illustrating how charge transfer from surface states bends the energy bands of p-type MAPbBr_3_. The built-in electric field (resulting from the existence of permanent charges) induces a spatial separation of the photocarriers on both sides of the space charge area. SCR: space charge region. E_F_: Fermi level. E_n_: surface states charge neutrality level. (b) Schematic representation illustrating the photocarrier generation and spatial separation by drift under the built-in electric field (step 1), the ion migration under illumination (step 2), the photocarrier recombination (step 3), and finally, the reverse ion migration under dark conditions (step 4). Note that the whole crystal volume is not represented in this sketch, which depicts only the processes occurring near the surface in the space charge area. (c) Plot of the surface potential as a function of time during an illumination sequence (λ = 515 nm, *P*_opt_ = 65.54 mW·cm^−2^). The curve has been rescaled by shifting the *y*-values in such a way that the SP at *t* = 0 is equal to 0 mV. The timing of the four steps depicted in b) is highlighted in c) by numbered circles.

The fast surface photovoltage polarity implies that negative charges accumulate quickly under illumination beneath the surface of the single crystal. This observation is fully consistent with the results of recent work by et Liu et al., who proposed [15] that a downward band bending occurs at the surface of p-type MAPbBr_3_ crystals. This p-type conductivity has been documented by numerous studies on MAPbBr_3_ thin films [26–27] and single crystals [28]. Here, the band bending is due to the existence of surface states which are filled by forming a charge-depleted layer (also called a space-charge layer) beneath the surface [15]. The resulting internal built-in electric field induces a spatial separation of the photogenerated carriers of opposite sign in the space charge region (Figure 3a). On the other hand, the opposite polarity of the slow SPV component implies that charge redistribution occurs in the system within a few seconds, which is highly likely to originate from photoinduced ion-migration mechanisms. As mentioned above, there is nowadays overwhelming evidence that hybrid perovskites should be treated (at least to some extent) as mixed electronic–ionic semiconductors [29]. Ion migration occurs in these materials due to the existence of anion and cation vacancies [30] and is already known to induce changes in the surface potential recorded by KPFM at time scales ranging from seconds to minutes [6,8–9]. Here, we assume that the excess of negative photocarriers at the surface attracts methyl ammonium cations (while bromide anions are repelled from the surface), resulting in an effective reduction of the surface photovoltage after a few seconds. In that time frame, the difference observed between the SP curves acquired during the initial and subsequent illumination sequences may indicate that the ion-migration process is not fully reversible (at least at the scale of the time interval between the illumination sequences). However, we also note that the surface potential returns fully to its initial value after the first illumination sequence. A plausible scenario (yet to be definitely confirmed) would be that negative charge carriers remain trapped in the space charge area with a counter cation partner, resulting in a neutral electrostatic balance before and after the first illumination pulse. Actually, the return to the dark state occurs most likely through a two-step process involving firstly the photocarriers, and secondly, the ionic species. Indeed, a closer look at the SP(*t*) curves in Figure 2a reveals that the SP is slightly more positive than initially just after switching the light pulse off. This SP overshoot becomes much more pronounced at higher fluence (as shown in Figure 3c and Figure S3d in Supporting Information File 1). Our interpretation is that most of the photocarriers recombine quickly after the pulse extinction, leaving an excess of positive cations near the surface. Then, a reverse migration of the cations towards the bulk occurs at a slower time scale (however, some of the cations remain eventually trapped with a counter electric charge after the first illumination sequence as suggested above). A schematic representation illustrating the full sequence of photocarrier generation and spatial separation, the ion migration, the photocarrier recombination, and finally, the reverse ion migration is given in Figure 3b.

The picture which emerges from the above discussion is remarkably consistent with the conclusions that can be drawn from former KPFM works performed on perovskite single crystals (band bending induced accumulation of negative photocarriers at the surface of MAPbBr_3_) and thin films (SPV time evolution resulting from an interplay between photocarrier and ionic species). Equally remarkable is the simultaneous observation of a photostrictive response very similar to the one reported from AFM measurements performed on MAPbI_3_ single crystals [16]. In particular, contrary to the conclusions of recent work based on Raman spectroscopy measurements [17], our data demonstrate that the crystal lattice also undergoes a dilatation under illumination in the case of the bromide compound. As shown hereafter, the photostrictive response displayed by our MAPbBr_3_ single crystal is moreover comparable in magnitude with the one reported by Zhou et al. for MAPbI_3_ in its cubic phase [16].

**Figure 4:**
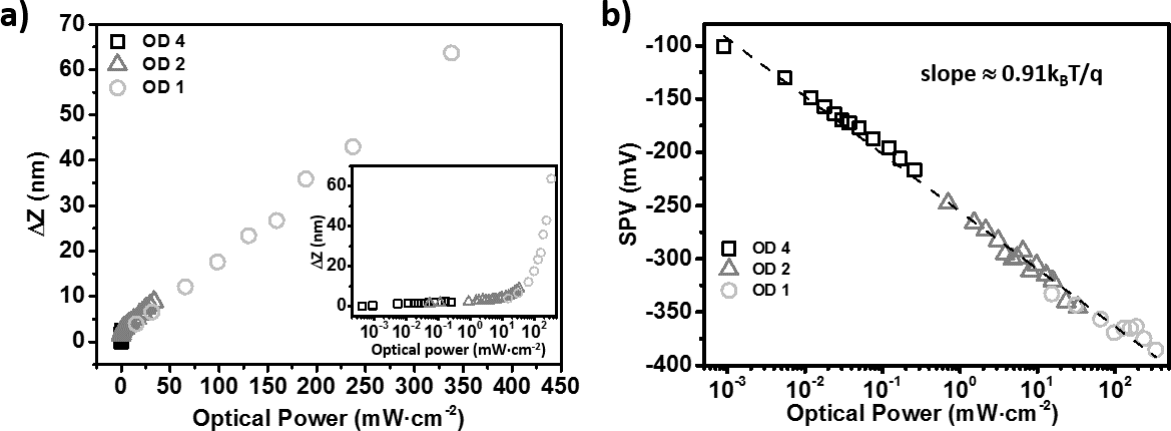
(a) Photostrictive signal as a function of the optical power at λ = 515 nm. Inset: plot in semi-logarithmic scale. (b) Fast component of the surface photovoltage as a function of the optical power (plot in semi-logarithmic scale) at λ = 515 nm. The slope of the linear fit (dotted line) is equal to ≈23.5 mV (calculation performed with the natural logarithm).Three optical densities (OD 1, 2 and 4) have been used to sweep the optical power over more than five decades (the data plotted with squares, triangles and circles have been acquired by using OD 4, OD 2 and OD 1 optical density filters, respectively).

To carry out a quantitative comparison, it is mandatory to analyze the light intensity dependence on the photostrictive effect. Here, the crystal photostriction is defined as the “fast component” of the height change under illumination (see Figure S3c, Supporting Information File 1). The height change appears in first analysis proportional to the light intensity (see Figure 4a) and displays no saturation up to ≈350 mW/cm^2^ under monochromatic illumination at 515 nm.The relative height change (i.e., height change divided by the sample thickness, here approximately 1 mm) under 100 mW/cm^2^ is equal to 18 ppm. This last value is remarkably close to the one reported [16] for the cubic phase of MAPbI_3_ (keeping in mind that our measurements are not performed under white light illumination, contrary to that reported for MAPbI_3_ single crystals). However, we note that the photostriction does not scale perfectly linearly with the fluence over the full measurement range. The photostriction data acquired in the low fluence regime (i.e., for optical powers below 10 mW/cm^2^) strongly deviates from a linear function, as clearly shown by the semi-logarithmic plot (inset in Figure 4a).

Further insight on the crystal photoresponse can be gained by analyzing the dependence of the SPV as a function of the optical power (Figure 4b). The fast component of the SPV displays a logarithmic dependence as a function of the illumination intensity. In principle, the slope of this curve (calculated with a natural logarithm) should fall between *k*_B_*T*/*q* and 2*k*_B_*T*/*q* (where *k*_B_*T*/*q* is the thermal voltage) depending of the strength of trap-delayed recombination processes [31]. A pure bimolecular recombination process cannot explain the anomalously low value deduced from our measurements. Such deviations have already been observed in small-molecule bulk-heterojunction solar cells [32], and have been recently explained by considering the contribution of interface recombination processes [33]. More precisely, this recent model predicts that slopes lower than the thermal voltage can be observed in the presence of surface recombination for systems where the bulk recombination is purely (or almost completely) bimolecular. This scenario is remarkably consistent with our previous deduction about the existence of surface states (which are here a key ingredient at the origin of the built-in electric field and photocarrier spatial separation), and with the fact that according to the literature [11] the trap density level should be quite low in the bulk of the perovskite single crystals.

**Figure 5:**
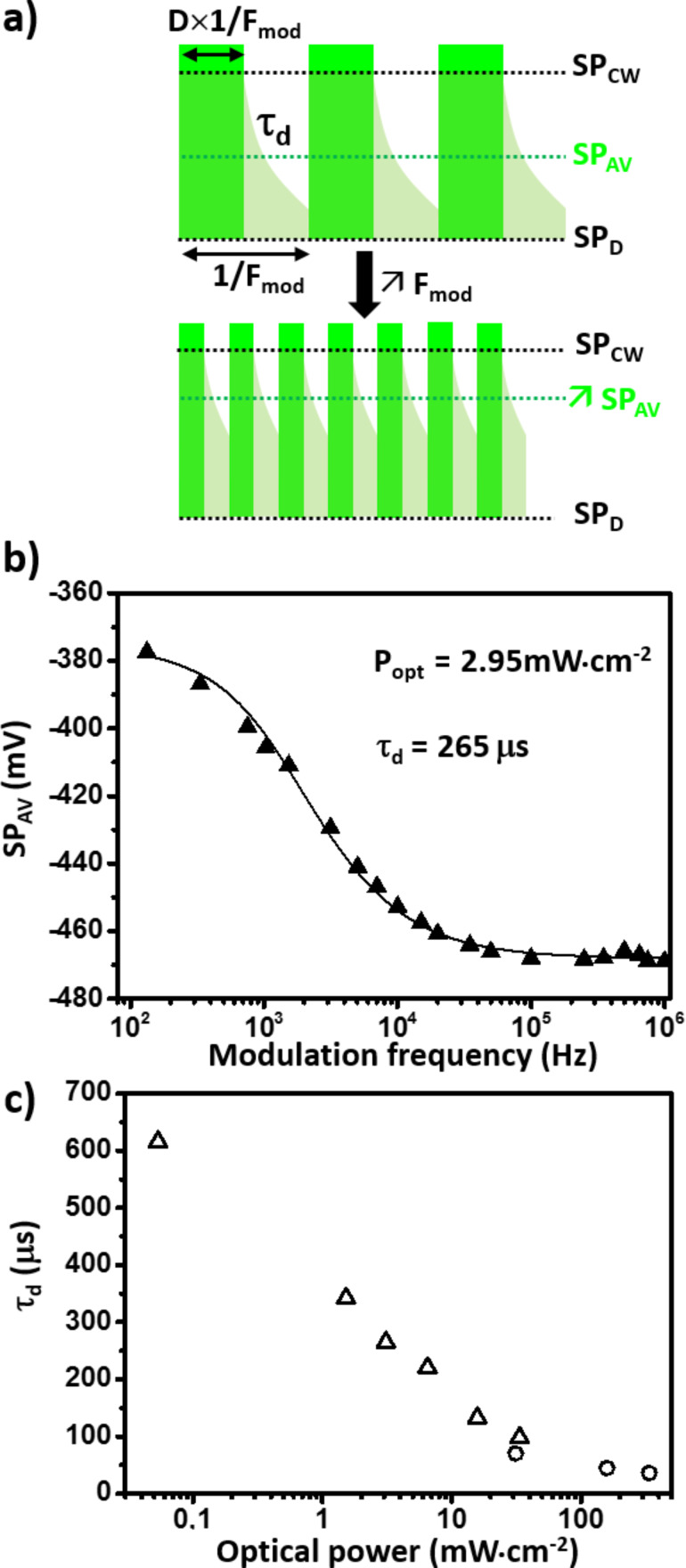
(a) Scheme of the surface potential time response under frequency modulated illumination. The SPV decay dynamics (characterized by a time constant τ_d_) determine the frequency evolution of the average potential, SP_av_, probed by KPFM. SP_D_ and SP_CW_ represent the in-dark surface potential and the maximum surface potential that would be measured under continuous wave illumination. Note that in this scheme the surface photovoltage (SPV = SPcw − SP_D_) is positive; in the case of our experiment, it displays an opposite polarity. (b) Experimental curves of the average surface potential as a function of the illumination modulation frequency F_mod_ acquired at 515 nm with an optical peak power of 2.95 mWcm^−2^. The result of the numerical fit performed to extract the SPV decay time constant is displayed by a solid line. (c) Plot of the SPV decay time constant as a function of the optical power.

To probe the photocarrier dynamics, an alternative approach consists of performing the KPFM measurements under frequency modulated illumination (Figure 5a). In the last years, several works have indeed shown that the effective carrier lifetime in photovoltaic thin films can be quantified by analyzing the dependence of the time-averaged surface potential (SP_av_) with respect to the modulation frequency (*f*) of the illumination source [20,34–36]. In short, if the system is characterized by a single photopotential decay process in the dark state (related to the photocarrier recombination), SP_av_ will increase with the modulation frequency and saturate when the time between the pulses becomes shorter than the photopotential time decay. If one uses simple exponential functions characterized by a unique time constant τ_d_ to account for the SPV decay between the light pulse, SPV_av_(*f*) curves can be fitted by Equation 1 [20]:

[1]
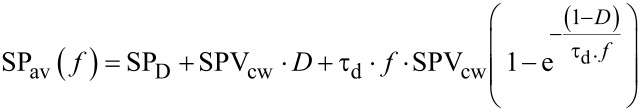


where SP_D_ is the “in-dark” surface potential, SPV_CW_ the surface photovoltage that would be measured under continuous wave illumination (which is equal to SP_av_ in the high frequency limit) and *D* is the illumination duty ratio.

As seen in Figure 5b the agreement between this fitting law and the experimental curves acquired on the MAPbBr_3_ single crystal is excellent, which confirms that the SPV dynamics can be properly accounted for on the basis of a single time constant decay. In addition, the time-resolved measurements have been carried out as a function of the fluence. As expected, the increase in charge carrier density (for increasing optical powers) leads to a decrease in the decay time (Figure 5c). More precisely, τ_d_ displays a linear decrease as a function of the optical power when plotted in semi-logarithmic scale in the 50 μW/cm^2^ to 20 mW/cm^2^ range. Although FMI-KPFM measurements are not performed in a nonperturbative regime [20], this observation seems consistent with former results of macroscopic transient photovoltage (TPV) measurements reported for MAPb(I_1−_*_x_*,Br*_x_*)_3_ perovskite thin films [37]. Besides, the similarity with the decay time values obtained by TPV measurements on MAPbI_3_ single crystals [38] is remarkable (e.g., τ_d_ = 175 μs under 10 mW/cm^2^ illumination for FMI-KPFM measurements on MAPbBr3, and τ_d_ = 234 μs under 0.1 sun in TPV measurements on MAPbI_3_).

Strikingly, the carrier lifetime and photostriction curves display slope changes occurring in the same optical power range (to ease the comparison, these data are presented side by side with the photostriction curve in log–log scale in Figure S5, Supporting Information File 1). For a fluence greater than a few 10 mW/cm^2^, the effective carrier lifetime decreases, and indeed less steeply when raising the optical power, while the photostriction displays an opposite trend. This photostrictive behavior indicates that the photocarrier coupling with the lattice becomes somehow “more efficient” in the high carrier density regime. It remains however difficult at this stage to draw a definitive conclusion about the origin of the photocarrier lifetime evolution in the high fluence regime. More specifically, the difficulty here is that the carrier density (in first approximation inversely proportional to the illumination intensity) remains a hidden parameter that cannot be directly deduced from our data (contrary to conventional macroscopic measurements where transient photovoltage can be combined with charge extraction [37]).

Here, we stress that the carrier recombination dynamics in the bulk may strongly differ from the SPV decays probed by time-resolved KPFM. Let us remind the reader here that the SPV originates from spatially separated carriers due to the existence of a built-in electric field at the vicinity of the surface. In the future, it would be highly desirable to quantify the vertical extension of the space charge area at the origin of the spatial separation of the photocarrier and to check how it compares with the light absorption depth, and more importantly, with the photocarrier diffusion length. Regarding the photostrictive effect, it has been indeed argued that the responsive layer is much thicker that the light penetration depth due to the diffusion of photocarriers in the bulk [16]. In turn, the SPV (more precisely its fast component) originates from the contributions of oppositely charged photocarriers located on either side of the space charge region. Thus, the photostriction signal may originate from a much thicker part of the crystal beneath the surface than the SPV.

Further experiments are in progress to map two-dimensional images of the SPV decay. Revealing the existence (or observing the absence) of time-decay contrasts related to surface (or subsurface) defects could help in assessing the relative weight of photocarriers localized near the surface and deeper in the bulk to the SPV recombination dynamics.

## Conclusion

In summary, we presented the results of a study intended to test if the optomechanical and optoelectronic properties of hybrid organic–inorganic perovskite single crystals can be investigated simultaneously by nc-AFM and KPFM. We successfully demonstrated that the height change and the surface potential shift under illumination originate from the crystal photostriction and the contributions of photogenerated charge carriers, respectively. The measurements revealed that, similar to the case of methylammonium lead triiodide, the photostrictive response of MAPbBr_3_ consists of a lattice expansion. Moreover, we have shown that our methodology based on the acquisition of spectroscopic curves in the time domain allows disentangling the contributions of the photocarriers to the surface photovoltage from the ones due to the light-induced migration of ionic species. Lastly, the effective carrier lifetime has been quantified by analyzing the dependence of the surface potential as a function of the frequency modulation of the illumination source. Thus, it has been possible to analyze both the photostriction and carrier lifetime as a function of the optical power. Our multimodal approach opens up new possibilities to investigate a wide range of photo-physical process and dynamical phenomena in organic–inorganic perovskites and related materials.

## Supporting Information

File 1 Additional experimental data.
Surface potential time evolution recorded during several successive illumination sequences. Measurements of the cantilever frequency shift as a function of the optical power and of the *z* variation as a function of the frequency shift set point. Curves of the relative height and surface potential recorded during illumination sequences on a highly oriented pyrolytic graphite substrate and on the MAPbBr_3_ single crystal for various optical powers. Photostrictive response and fast component of the surface photovoltage as a function of the optical power for 685 nm, 515 nm and 405 nm illumination. Curves of the SPV time decay and photostriction as a function of the optical power.
