# Supplementary material for: Multimodal noncontact atomic force microscopy and Kelvin probe force microscopy investigations of organolead tribromide perovskite single crystals
Source: Beilstein J Nanotechnol. 2018 Jun 7;9:1695–704. doi: 10.3762/bjnano.9.161 (PMC6009450; doi:10.3762/bjnano.9.161)
Supplement: File 1 — Additional experimental data. Surface potential time evolution recorded during several successive illumination sequences. Measurements of the cantilever frequency shift as a function of the optical power and of the z variation as a function of the frequency shift set point. Curves of the relative height and surface potential recorded during illumination sequences on a highly oriented pyrolytic graphite substrate and on the MAPbBr3 single crystal for various optical powers. Photostrictive response and fast component of the surface photovoltage as a function of the optical power for 685 nm, 515 nm and 405 nm illumination. Curves of the SPV time decay and photostriction as a function of the optical power. [file Beilstein_J_Nanotechnol-09-1695-s001.pdf]

Supporting Information

for

Multimodal noncontact atomic force microscopy and Kelvin probe force microscopy investigations of organolead tribromide perovskite single crystals

Yann Almadori[1], David Moerman[2], Jaume Llacer Martinez[2], Philippe Leclère[2], and Benjamin Grévin[*1]

Address: [1]Université Grenoble Alpes, CNRS, CEA, INAC-SyMMES, 38000 Grenoble, France, and [2]Laboratory for Chemistry of Novel Materials, Center of Innovation and Research in Materials & Polymers (CIRMAP), University of Mons, Place du Parc 20, B7000 Mons, Belgium

Email: Benjamin Grévin* - benjamin.grevin@cea.fr

* Corresponding author

Additional experimental data

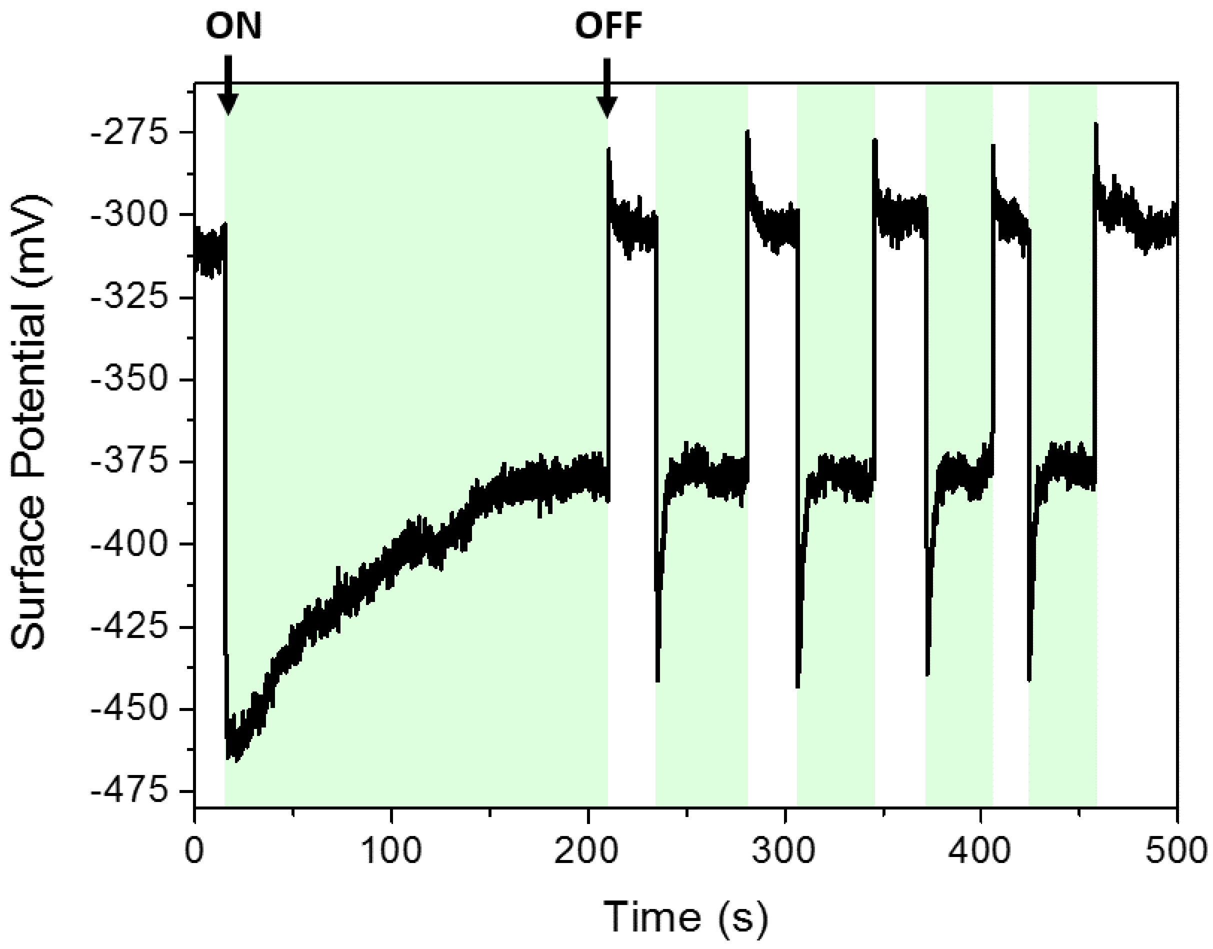

Figure S1: Plot of the KPFM surface potential as a function of time during five successive illumination sequences (λ = 515 nm, P_{opt} = 2.95 W/cm^2).

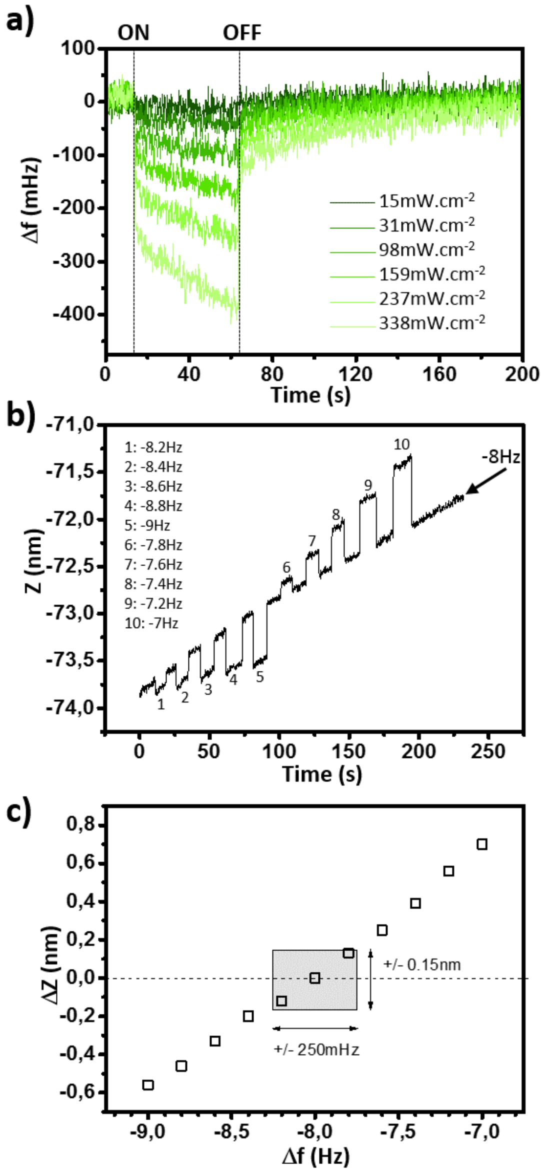

Figure S2: (a) Plots of the cantilever frequency shift as a function of time recorded during illumination sequences with increasing optical powers. The fast photo-response of the frequency shift is at maximum equal to 250 mHz.NB: these measurements have been performed with the tip kept at a fixed position 1μm away from the sample surface. **(b)** Tip height as a function of time. The frequency shift set-point (-8Hz for the line base) is changed while keeping the z-regulation active. **(c)** Relative height change as a function of the frequency shift setpoint deduced from the data presented in b). From these data, we estimate that a frequency detuning of 250 mHz shall induce a z-artefact of 0.15 nm.

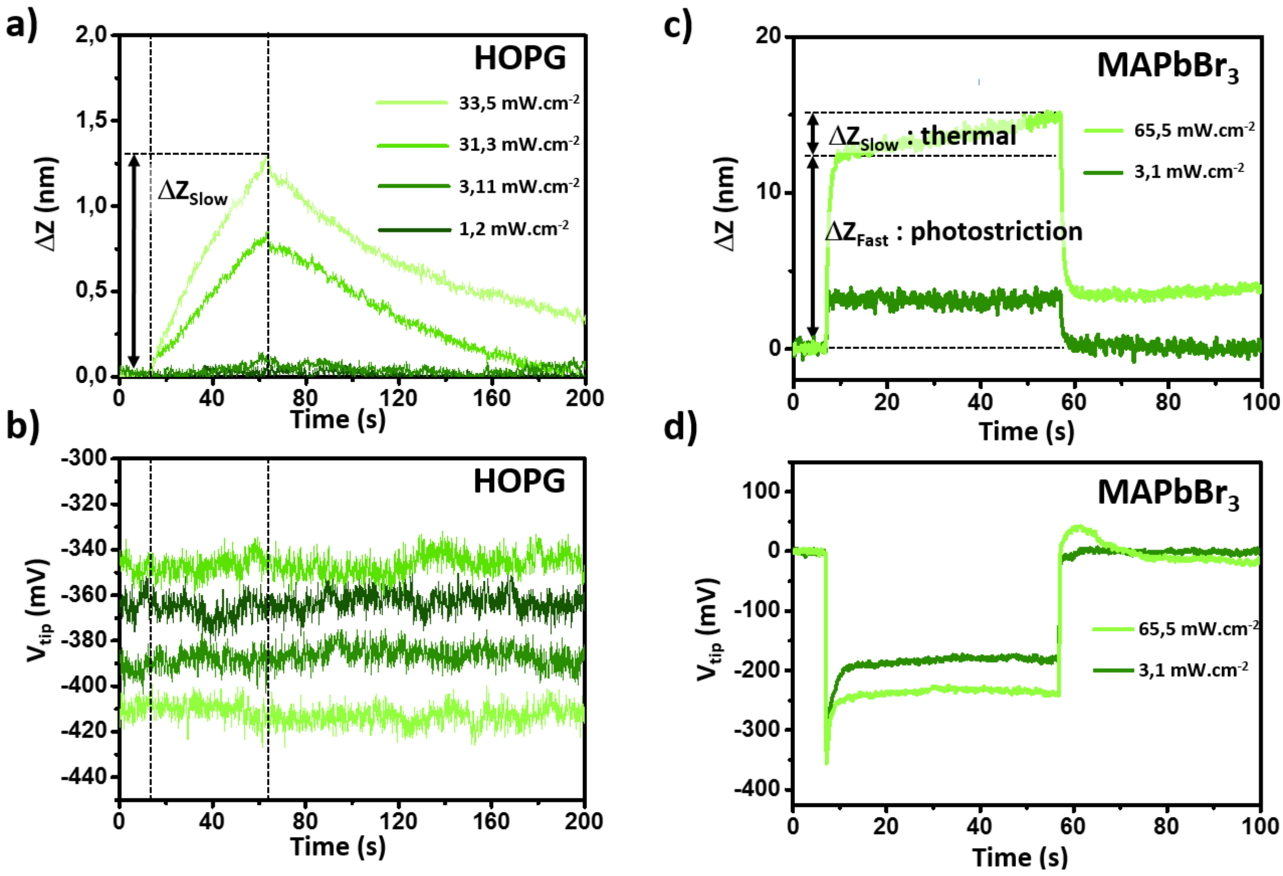

Figure S3: (a,b) Plots of the tip height change relative to its initial position (a) and of the KPFM surface potential (b) and as a function of time during illumination sequences (λ = 515 nm) performed with 4 different optical powers on an HOPG substrate. (c,d) Plots of the tip height change relative to its initial position (c) and of the KPFM surface potential (d) and as a function of time during illumination sequences (λ = 515 nm) performed with 2 different optical powers on the $MAPbBr_3$ single crystal. The curves of the surface potential have been normalized by shifting the y-values in such a way that the SP at t = 0 is equal to 0 mV.

.

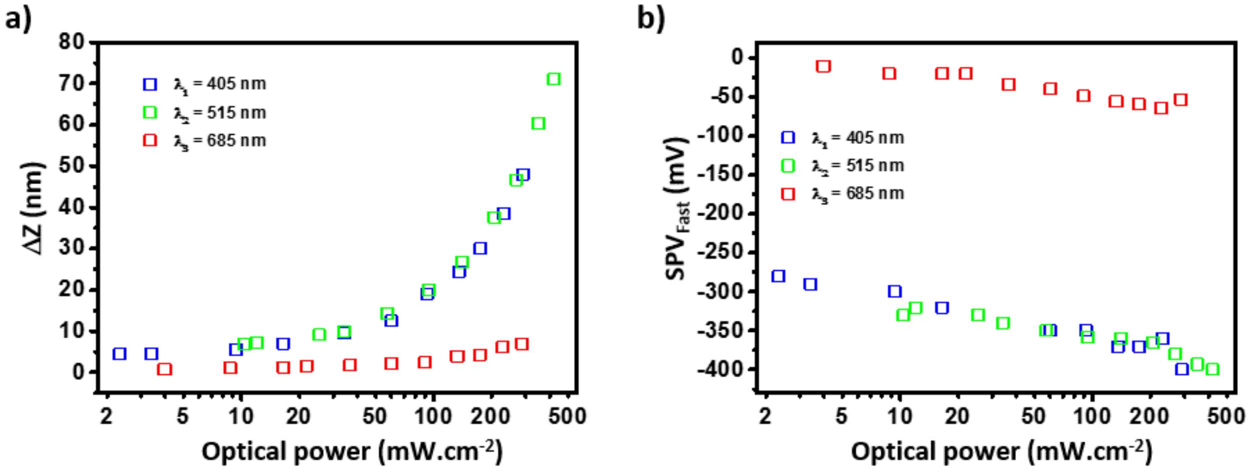

Figure S4: (a,b) Photostrictive signal (a) and fast component of the surface photovoltage (b) as a function of the optical power for three different wavelength.

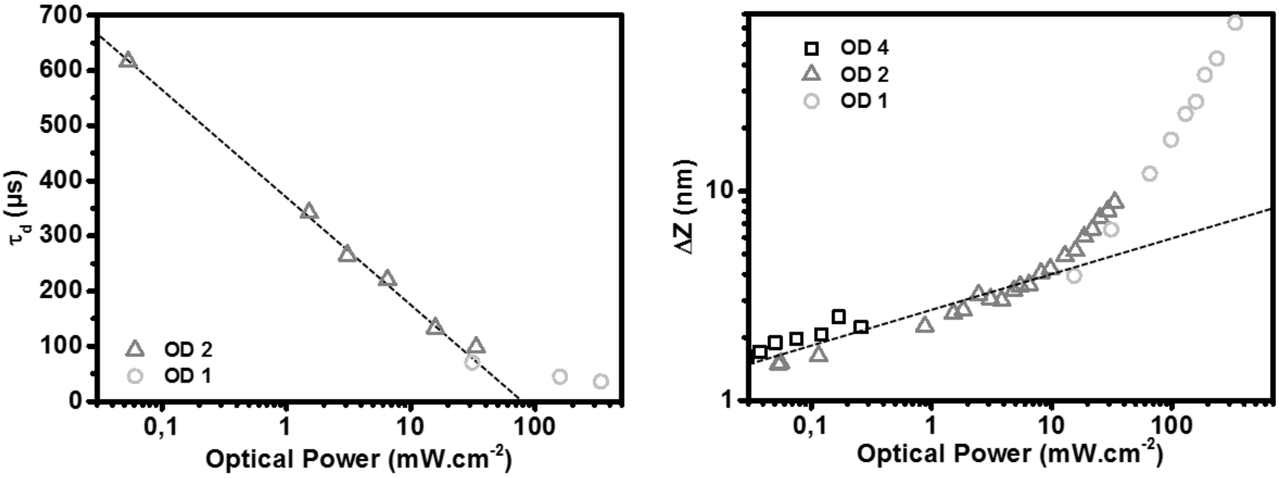

Figure S5: (a) SPV decay time constant measured by FMI-KPFM as a function of the optical power. (b) Photostrictive signal as a function of the optical power in log-log scale. λ = 515 nm. The dotted lines in a) and b) are only guidelines for the eye.
